# Molecular identification and antifungal susceptibility profiles of *Candida dubliniensis* and *Candida africana* isolated from vulvovaginal candidiasis: A single‐centre experience in Iran

**DOI:** 10.1111/myc.13280

**Published:** 2021-05-03

**Authors:** Gholamreza Shokoohi, Javad Javidnia, Hossein Mirhendi, Athar Rasekh‐Jahromi, Ali Rezaei‐Matehkolaei, Saham Ansari, Faeze Maryami, Sahand Goodarzi, Orazio Romeo

**Affiliations:** ^1^ Department of Medical Parasitology and Mycology School of Medicine Jahrom University of Medical Sciences Jahrom Iran; ^2^ Zoonosis Research Center Jahrom University of Medical Sciences Jahrom Iran; ^3^ Student Research Committee Center Mazandaran University of Medical Sciences Sari Iran; ^4^ Department of Medical Mycology School of Medicine Mazandaran University of Medical Sciences Sari Iran; ^5^ Department of Medical Parasitology and Mycology School of Medicine Isfahan University of Medical Sciences Isfahan Iran; ^6^ Department of Obstetrician and Gynecology Jahrom University of Medical Sciences Jahrom Iran; ^7^ Infectious and Tropical Diseases Research Center Health Research Institute Ahvaz Jundishapur University of Medical Sciences Ahvaz Iran; ^8^ Department of Parasitology and Mycology School of Medicine Shahid Beheshti University of Medical Sciences Tehran Iran; ^9^ Department of Chemical, Biological, Pharmaceutical and Environmental Sciences University of Messina Messina Italy

**Keywords:** *Candida africana*, *Candida dubliniensis*, efinaconazole, *HWP1* gene, luliconazole, vulvovaginal candidiasis

## Abstract

**Background:**

Vulvovaginal candidiasis (VVC) is a common and debilitating long‐term illness affecting million women worldwide. This disease is caused mainly by *Candida albicans* and a lesser extent by other species, including the two phylogenetically closely related pathogens *Candida africana* and *Candida dubliniensis*.

**Objectives:**

In this study, we report detailed molecular epidemiological data about the occurrence of these two pathogenic yeasts in Iranian patients affected by VVC, or its chronic recurrent form (RVVC), and provide, for the first time, data on the antifungal activity of two new drugs, efinaconazole (EFN) and luliconazole (LUL).

**Methods:**

A total of 133 vaginal yeast isolates, presumptively identified as *C* *albicans* by phenotypic and restriction analysis of rDNA, were further analysed by using a specific molecular method targeting the *HWP1* gene. All *C* *africana* and *C* *dubliniensis* isolates were also tested for their in vitro susceptibility to a panel of modern and classical antifungal drugs.

**Results and Conclusions:**

Based on the molecular results, among 133 germ‐tube positive isolates, we identify 119 *C* *albicans* (89.47%), 11 *C* *africana* (8.27%) and 3 *C* *dubliniensis* (2.26%) isolates. *C* *africana* and *C* *dubliniensis* showed low MIC values for most of the antifungal drugs tested, especially for EFN and LUL, which exhibited a remarkable antifungal activity. High MIC values were observed only for nystatin and terbinafine. Although *C* *albicans* remains the most common *Candida* species recovered from Iranian VVC/RVVC patients, our data show that its prevalence may be slightly overestimated due to the presence of difficult‐to‐identify closely related yeast, especially *C* *africana*.

## 1 INTRODUCTION

Vulvovaginal candidiasis (VVC) is one of the most common and widespread human fungal infection in the world that affects 70%–75% of women at least once in their lifetime, especially in childbearing age.^1^ According to recent estimates,^2^ up to 9% of young women experience recurrent episodes (4 or more) of VVC (RVVC) each year, which result in hundreds of millions of people affected globally, and in a huge economic loss estimated to be in tens of billions of dollars.^2^

The infection is caused by some species belonging to *Candida* genus (phylum *Ascomycota*; sub‐phylum *Saccharomycotina*; order: *Saccharomycetales*), a complex and heterogeneous group of yeast‐like fungi widely distributed in nature.^3^ This genus contains at least 300 taxa^4^ but, of the over 40 species known as etiological agents of candidiasis,^5^ only 5 (*Candida albicans*, *Candida* *glabrata*, *Candida* *tropicalis*, *Candida* *parapsilosis* and *Candida* *krusei*) are responsible for more than 95% of the infections occurring in humans.^6^

*C albicans* is the most pathogenic species of the group and remains the most frequently isolated yeast from clinical specimens, including vaginal swabs.^7^, ^8^ In fact, over 90% of VVC cases are attributable to this species while the remaining ones are caused by several other species, such as *C glabrata*, *C tropicalis* and *C parapsilosis*, which are collectively referred to as non‐*albicans Candida* species.^8^

Until 1995, *C* *albicans* was considered the only germ‐tube and chlamydospores positive *Candida* species infecting humans, but molecular comparative studies, performed on some atypical Irish *C* *albicans* strains, revealed the existence of high levels of genetic diversity which led to the description of a new phylogenetically related species, called *Candida dubliniensis*.^9^ In the same year, other unusual germ‐tube positive *C* *albicans* isolates were recovered from African patients with vaginitis,^10^ and subsequently proposed as representatives of a novel species, *Candida africana*,^11^ on the basis of some unusual phenotypes clearly different from those of typical *C* *albicans* isolates, for example the lack of chlamydospores production.^12^ However, although the status of new species has been genetically confirmed for *C* *dubliniensis*, for *C* *africana* the taxonomic position is still controversial^13^ and some authors consider it as a biovar of *C albicans*.^12^ Nevertheless, *C* *africana* represents undoubtedly one the most intriguing evolutionary lineage within the global *C* *albicans* population^14^, ^15^ and recent genome‐wide studies^16^, ^17^ have tried to clarify its evolutionary history by drawing an interesting and fascinating picture on the origin and evolution of the members of the *C* *albicans* clade, including the well‐known and synonymised *Candida* *stellatoidea*.^13^ Interestingly, these studies suggested that *C* *albicans* probably originated from an ancestral hybridisation event between two divergent lineages, which also generated *C* *africana* and *C* *stellatoidea* making their taxonomic position in the clade even more uncertain and complicated.^13^ The clinical and epidemiological implications of these findings are very important because members of the *C* *albicans* clade, as well as the non‐hybrid species *C* *dubliniensis*,^17^ despite exhibiting some lineage‐specific phenotypes, they may still be misidentified as the same species by conventional mycological methods.^12^, ^13^ Furthermore, the paucity of specific drug susceptibility data for these genetically distinct *Candida* lineages makes it difficult to choose the appropriate therapeutic treatment for VVC/RVCC caused by them and contributes to the increase of azole‐resistant strains.^18^

Although *C* *africana* and *C* *dubliniensis* are widely distributed across the world,^9^, ^15^, ^19^ there are limited data on the extent of RVVC caused by these pathogenic yeasts in Iranian women.^20^ Recent studies have shown that most of vaginal *C* *africana* isolates from Iran exhibit resistance to various antifungal drugs,^21^, ^22^ and ~28% of world isolates appear to be resistant to itraconazole.^23^ Furthermore, although *C* *africana* is generally associated with vaginal infections, in Iran, this pathogen has also been reported as a cause of paediatric candiduria,^24^ and/or oropharyngeal candidiasis in cancer patients.^25^ From these latter patients, fluconazole‐ and amphotericin B‐resistant isolates have been also recovered.^25^ These epidemiological findings support the hypothesis that *C* *africana* prevalence, and/or drug resistance, can be geographically variable^23^ and suggest further studies, in particular in vitro susceptibility testing of promising new drugs.

The aim of this study was to evaluate the prevalence of *C* *africana* and *C* *dubliniensis* in a large cohort of Iranian patients with VVC/RVVC and to investigate the susceptibility of the isolates toward two new azoles, luliconazole (LUL) and efinaconazole (EFN), in comparison with other classical antifungal drugs.

## 2 MATERIALS AND METHODS

### 2.1 Patients and yeast isolates

This research was performed on 295 vaginal swab samples, collected during 1‐year period (August 2018–September 2019) from Iranian patients presenting signs and symptoms of VVC who referred to clinics of gynaecology in Jahrom city, south of Iran. Swabs were plated directly onto Sabouraud dextrose agar plates containing chloramphenicol (50 mg/L) and then incubated at 37°C for 48–72 h. Before molecular identification, all germ‐tube positive *Candida* isolates were subcultured on the chromogenic medium CHROMagar *Candida* (CHROMagar), at 35°C for 48 h, to ensure isolation of pure colonies and/or recognition of mixed culture.^26^ This study was approved by the Ethics Committee of Jahrom University of Medical Sciences (n° IR.JUMS.REC.1398.001).

### 2.2 Genomic DNA extraction, molecular identification and partial *HWP1* gene sequencing

In this study, a preliminary molecular screening of the yeast isolates was accomplished using a polymerase chain reaction‐restriction fragment length polymorphism (PCR‐RFLP) analysis of ribosomal internal transcribed regions (ITS).^27^, ^28^

Total genomic DNA was extracted from each isolate using the glass‐bead phenol‐chloroform method according to a previous study.^29^ Purified DNA samples were then subjected to PCR‐RFLP analysis as previously described in Gharaghani et al.^27^ Briefly, the ITS‐rDNA regions were amplified using 0.5 μM of the fungal universal primers ITS1 and ITS4, 4 μl of DNA template, 12.5 μl of 2× Taq DNA master mix (Ampliqon) and PCR‐grade water to make a final reaction volume of 25 μl.^30^ PCR cycles included initial denaturation at 94°C for 5 min, followed by 35 cycles of denaturation at 94°C for 30 s, annealing at 58°C for 30 s, extension at 72°C for 1 min and a final extension step of 7 min at 72°C. After PCR, 6 μl of each amplicon was digested for 2 h at 37°C in 15 μl of restriction mixture containing 1.5 μl reaction buffer, 7 μl water and 0.5 μl MspI as a restriction enzyme. Subsequently, the digested products were separated and visualised on a 2% (wt/vol) agarose gel stained with ethidium bromide (0.5 μg/ml) and photographed under ultraviolet light at 305 nm. Final species identification was done by comparing the obtained RFLP patterns with those of reference strains used in other studies.^27^, ^28^

In order to correctly identify each member of the *C* *albicans* clade, all isolates were further characterised by using a simple PCR‐based assay developed for rapid and specific identification of these pathogens.^12^, ^31^ This method is based on partial amplification of the *HWP1* gene using only a single pair of primers: CR‐f‐GCTACCACTTCAGAATCATCATC‐3′ and CR‐r‐GCACCTTCAGTCGTAGAGACG.^31^ The reaction mixture and conditions used for in vitro amplification were the same as those previously described by Romeo and Criseo, 2008.^31^ PCR products were separated on a 1.3% (wt/vol) agarose gel and, according to the size of the amplicon produced, *Candida* isolates were classified as follows: *C* *albicans* (~941 bp), *C* *stellatoidea* (~800 bp), *C* *africana* (~740 bp) and *C* *dubliniensis* (~569 bp).^12^, ^31^

All *HWP1* fragments obtained from isolates identified as *C* *africana* and *C* *dubliniensis*, including three random selected *C* *albicans* isolates, were also sequenced using the same CR‐f/CR‐r primer set mentioned above. PCR products were sequenced via the ABI PRISM BigDye Terminator Cycle Sequencing Ready Reaction Kit (Applied Biosystems) on an automated ABI PRISM^TM^ 3730 DNA sequencer (Applied Biosystems), following the manufacturer's guidelines. The resulting electropherograms were visually inspected and compared with the corresponding *HWP1* gene reference sequences of *C* *albicans* (GenBank: XM_704869.2), *C* *dubliniensis* (GenBank: XM_002419949.1) and *C* *africana* (GenBank: EU477610.1).

All the nucleotide sequences obtained in this study have been deposited in the GenBank database under the following accession numbers: MT361747‐MT361763.

**FIGURE 1:**
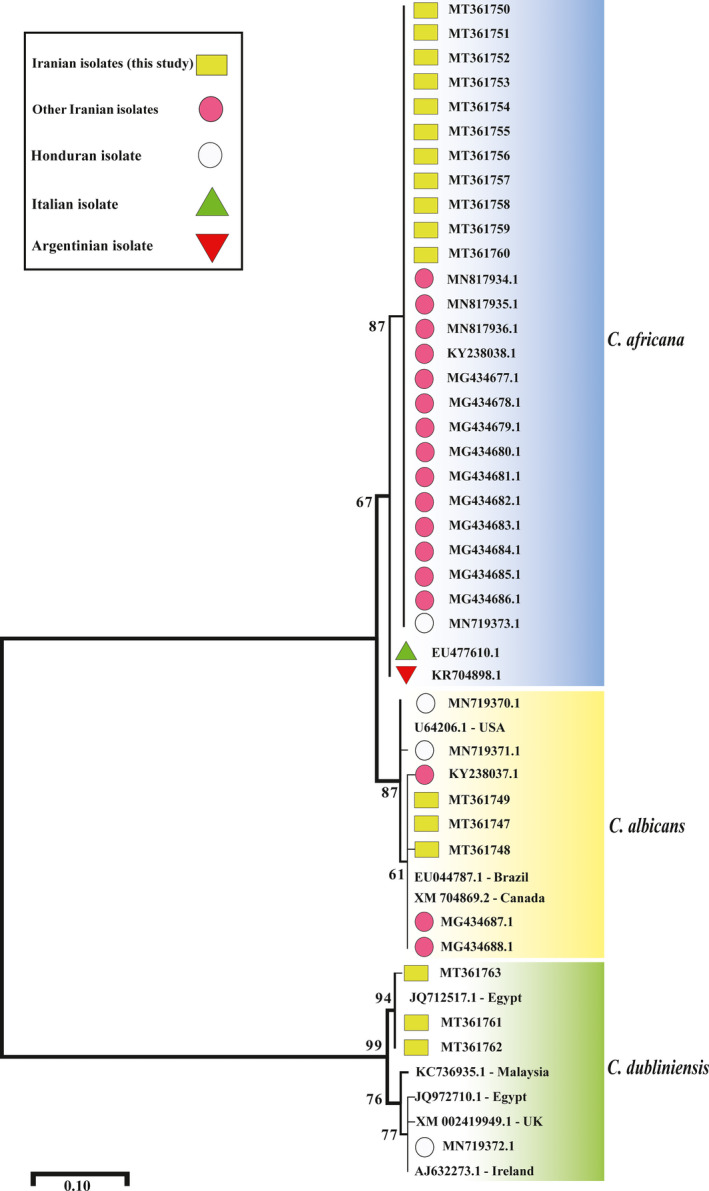
Phylogenetic analysis of *C albicans*, *C* *africana* and *C* *dubliniensis* based on *HWP1* gene sequences obtained in this and other studies. The GenBank accession numbers for each sequence used in the analysis are indicated in the tree. The evolutionary history was inferred using the maximum‐likelihood method based on the Tamura–Nei substitution model. Numbers at the nodes represent bootstrap values expressed as percentages of 1000 replicates

For further in silico sequence analysis and phylogenetic reconstruction, additional *HWP1* sequences were retrieved from the GenBank database and included in this study (Figure 1). The maximum‐likelihood method was employed for phylogenetic analysis by using unambiguously aligned *HWP1* sequences based on the Tamura‐Nei substitution model as implemented in the MEGA software, version 7.^32^

### 2.3 Antifungal susceptibility testing

Evaluation of the in vitro susceptibility profiles of *C* *dubliniensis* and *C* *africana* to various antifungal drugs was performed according the CLSI M27‐A3 and M27‐S4 documents.^33^, ^34^ The susceptibility of the isolates to amphotericin B (AMB; Bristol‐Myers‐Squib), itraconazole (ITC; Janssen Research Foundation), voriconazole (VRC; Pfizer, Central Research), clotrimazole (CLO) and ketoconazole (KTO; Sigma) was tested in the concentration range of 0.016–16 μg/ml, whereas terbinafine (TRB), nystatin (NYS; Sigma) and fluconazole (FLU; Pfizer) activities were evaluated in the concentration range of 0.063–64 μg/ml.

The two new antifungals, LUL and EFN (Sigma), were tested in the concentration range of 0.008–8 μg/ml, whereas the range for caspofungin (CAS; Sigma) was 0.008–8 μg/ml.

The yeast inoculum suspensions were prepared spectrophotometrically at 530 nm to a percent transmission in the range 75–77. Standardised inocula were in the range of 0.5–2.5 × 10^3^ CFU/ml.

To estimate the minimum inhibitory concentration (MIC), the microdilution plates were incubated at 35°C and observed at 24 h. However, unlike typical *C* *albicans* isolates, *C* *africana* usually exhibits slower growth rates at different temperatures.^16^, ^35^ Therefore, if no growth was observed at 24 h, the incubation time was extended to 48 h. After incubation, MIC endpoints were determined visually by comparing the turbidity with the drug‐free growth control well. MIC was interpreted as the lowest drug concentration capable to visibly inhibiting fungal growth (ie inducing 100% inhibition in the case of amphotericin B and nystatin), or significantly reducing fungal growth (50% for all other antifungal agents), compared to the drug‐free control growth. *C* *parapsilosis* (ATCC 22019) and *C* *krusei* (ATCC 6258) were used as quality control strains.^33^, ^34^

## 3 RESULTS

Patient data and clinical characteristics of VVC/RVVC caused by *C africana* and *C* *dubliniensis* are summarised in Table 1. A total of 149 yeast isolates were recovered from 295 examined vaginal swabs. Of these, 133 (89.2%) were germ‐tube positive and grew as typical *C* *albicans* green colonies on CHROMagar *Candida*. As expected for germ‐tube positive *Candida* species,^28^ all 133 yeast isolates produced a 537 bp amplicon by ITS amplification and two smaller DNA fragments (239 and 298 bp) after digestion with MspI restriction endonuclease,^28^ confirming that the PCR‐RFLP method employed was not able to discriminate between *C* *albicans*, *C* *dubliniensis* and *C* *africana*.^28^ On the contrary, partial amplification of the *HWP1* gene yielded single DNA bands of different sizes allowing to correctly identify 119 *C* *albicans* (89.47%), 11 *C* *africana* (8.27%) and 3 *C* *dubliniensis* (2.26%) isolates. Sequencing of the *HWP1* amplicons of all Iranian *C* *africana*, *C dubliniensis* and 3 representative *C* *albicans* isolates confirmed the results obtained by PCR identification. In fact, all Iranian *C* *africana* isolates showed 100% sequence similarity with several other *HWP1* sequences from Iran and Honduras. More specifically, comparison of the 11 *C* *africana HWP1* sequences with 17 reference sequences available in GenBank (Iran *n* = 14, Italy *n* = 1, Honduras *n* = 1 and Argentina *n* = 1; Figure 1) showed a sequence similarity ranging from 99.72% to 100% indicating that *C. africana* isolates recovered from Italy (GenBank: EU477610.1) and Argentina (GenBank: KR704898.1) were genetically, slightly, different from the Iranian ones (Figure 1).

**TABLE 1:** Clinical characteristics of vulvovaginal candidiasis caused by *C* *africana* (1–11) and *C* *dubliniensis* (12–14) isolates in VVC/RVVC patients

Source	Patient no.	Age (years)	Signs and symptoms	Risk factors	Antifungal therapy	Outcome
RVVC	1	27	Itching, vaginal discharge, dysuria	Receipt of antibiotics	Oral fluconazole	Cured
	2	23	Itching, burning, vaginal discharge, pain during sexual activity	‐	Clotrimazole	Cured
	3	44	Itching, burning, vaginal discharge	Receipt of antibiotics	Clotrimazole	Cured
	4	34	Itching, dysuria, pain during sexual activity	Diabetes	Clotrimazole	Cured
VVC	5	33	Itching, burning, pain during sexual activity	‐	Clotrimazole	Cured
	6	38	Vaginal discharge, pain during sexual activity	‐	Clotrimazole	Cured
	7	57	Itching, burning, vaginal discharge	Hypertension	Clotrimazole	Cured
	8	40	Itching, burning	‐	Clotrimazole	Cured
	9	37	Itching, burning, vaginal discharge	‐	Clotrimazole	Cured
	10	36	Itching, burning, pain during sexual activity	‐	Clotrimazole	Cured
	11	30	Itching, burning	‐	Clotrimazole	Cured
	12	24	Itching, burning, vaginal discharge, soreness	Receipt of antibiotics	Clotrimazole	Cured
	13	44	Itching, burning, vaginal discharge, pain during sexual activity	‐	Clotrimazole	Cured
	14	32	Itching, burning, soreness	Receipt of antibiotics	Clotrimazole	Cured

Abbreviations: RVVC, recurrent vulvovaginal candidiasis; VVC, Vulvovaginal candidiasis.

Regarding *C* *dubliniensis*, Iranian isolates were highly similar among them with only one nucleotide substitution (nucleotide position n° 360) detected in 1 (GenBank: MT361763) of the 3 isolates recovered in this study. Interestingly, two isolates (GenBank: MT361761 and MT361762) showed 100% sequence similarity with an Egyptian *C. dubliniensis* isolate (GenBank: JQ712517) (Figure 1) but were genetically different from several other isolates recovered in diverse geographical areas (Figure 1). Figure 1 depicts a maximum‐likelihood phylogenetic tree obtained by using *HWP1* sequences from this study and other sequences currently available in the GenBank database.

The results of the in vitro antifungal susceptibility testing for *C* *africana* and *C* *dubliniensis* are shown in Table 2. The geometric mean MIC against all tested isolates was 0.019 and 0.024 μg/ml for the two novel drugs EFN and LUL, respectively (Table 2). However, in general, except for nystatin and terbinafine, all other antifungal agents tested showed low MIC values and therefore all the isolates were considered susceptible to these drugs (Table 2).

**TABLE 2:** In vitro susceptibilities of 14 *Candida* isolates against eleven antifungal agents

*Candida* species	Antifungal drugs	MIC Parameters (µg/ml)			
		Range	G mean^a^	MIC_50_	MIC_90_
All isolates (*n* = 14)	Amphotericin B	0.031–0.5	0.080	0.063	0.5
	Fluconazole	0.031–16	0.220	0.125	0.25
	Ketoconazole	0.031–2	0.118	0.063	0.125
	Clotrimazole	0.031–0.063	0.046	0.063	0.063
	Itraconazole	0.031–32	0.125	0.063	0.125
	Voriconazole	0.031‐16	0.1175	0.063	0.125
	Nystatin	16–32	21.926	32	32
	Caspofungin	0.031–0.063	0.040	0.031	0.063
	Luliconazole	0.008–0.063	0.024	0.016	0.031
	Efinaconazole	0.008–0.063	0.019	0.016	0.031
	Terbinafine	16–32	17.041	16	16
*C africana* ^b^ (*n* = 11)	Amphotericin B	0.031–0.5	0.062	0.063	0.25
	Fluconazole	0.031–16	0.194	0.125	0.25
	Ketoconazole	0.031–2	0.104	0.063	0.25
	Clotrimazole	0.031‐0.063	0.043	0.031	0.063
	Itraconazole	0.031–32	0.11	0.063	0.125
	Voriconazole	0.031–16	0.104	0.063	0.125
	Nystatin	16–32	24.87	32	32
	Caspofungin	0.031–0.063	0.040	0.031	0.063
	Luliconazole	0.008–0.063	0.022	0.031	0.031
	Efinaconazole	0.008–0.063	0.019	0.016	0.031
	Terbinafine	16–32	17.04	16	16
*C dubliniensis* ^c^ (n = 3)	Amphotericin B	0.063–0.5	0.126	ND	ND
	Fluconazole	0.125–0.25	0.157	ND	ND
	Ketoconazole	0.063–0.125	0.079	ND	ND
	Clotrimazole	0.063	0.063	ND	ND
	Itraconazole	0.063–0.125	0.079	ND	ND
	Voriconazole	0.063–0.125	0.079	ND	ND
	Nystatin	16–32	20.16	ND	ND
	Caspofungin	0.031–0.063	0.050	ND	ND
	Luliconazole	0.016–0.031	0.020	ND	ND
	Efinaconazole	0.008–0.016	0.013	ND	ND
	Terbinafine	16	16	ND	ND

Abbreviation: ND, Not Determined.

^a^ G mean: Geometric mean.

^b^ 48 h‐MIC values.

^c^ 24 h‐MIC values.

## 4 DISCUSSION

Vaginal candidiasis is still one of the most common human fungal infections worldwide that affects an estimated 138 million women each year, a number that is destined to increase by more than 20 million over the next 10 years.^2^ In Iran, over 2.7 million women, aged between 15 and 50 years, suffer from RVVC but current epidemiological data are not sufficient to establish with certainty the real burden of this disease in this country.^36^ However, in accordance with current global epidemiological trends,^2^, ^8^ *C* *albicans* is the most commonly isolated *Candida* species in Iranian VVC/RVVC patients,^20^, ^21^, ^22^, ^37^, ^38^, ^39^ even if our data confirm that its prevalence is slightly overestimated due to the presence of two difficult‐to‐identify closely related yeasts, *C* *africana* and *C* *dubliniensis*. These yeasts are often reported as members of the so‐called ‘*C* *albicans* species complex’ by many authors^22^, ^23^, ^28^, ^35^, ^40^, ^41^, ^42^, ^43^ but, according to recent changes in naming fungal species of medical interest,^44^ and the new developments in the phylogeny of the *C* *albicans* clade,^13^, ^17^ we believe that the denomination of a ‘species complex’ is no longer appropriate to indicate these pathogenic yeasts and therefore, according to Mixão et al, 2020,^13^ there is an urgent need to clarify the taxonomic position of *C* *albicans* and its related lineages.

Collectively, in this study, *C* *dubliniensis* and *C* *africana* make up 10.5% (14/133) of VVC/RVVC cases caused by germ‐tube positive *Candida* isolates and, in this context, *C* *africana* makes the most significant contribution (11/14 non‐*albicans* isolates; ~78.6%) which is in line with previous studies, including its high ability to cause mainly vaginal infections.^12^, ^15^, ^23^, ^35^, ^41^ Conversely, *C* *dubliniensis* has been frequently associated with oral infections in HIV‐positive patients,^9^, ^12^ and our data confirm its very low prevalence in VVC/RVVC patients as already evidenced by several previous Iranian and international studies.^12^, ^45^, ^46^, ^47^, ^48^, ^49^

*C africana* has a worldwide distribution^12^, ^15^, ^23^, ^41^ and a recent global epidemiological meta‐analysis by Gharehbolagh et al^23^ showed that within *C* *albicans*/*C* *africana*/*C* *dubliniensis* group its overall prevalence is estimated to be 1.67% with Iran and Honduras showing the highest global prevalence (~3%).^23^ Interestingly, in silico comparison of *HWP1* sequences and phylogenetic analysis showed that our *C* *africana* isolates were genetically identical to other isolates from Iran and Honduras (Figure 1), and slightly different from those of Italian and/or Argentinean origin. However, the lack of intraspecific genetic variation within Iranian *C* *africana* isolates agrees with recent studies showing that the global population structure of this *Candida* yeast is highly clonal,^16^ and therefore small genetic variations among isolates can be evidenced only by using techniques that allow to analyse a large fraction of DNA sequences or its entire genome.^15^, ^16^

Our study reports one of the largest clusters of *C* *africana* isolates from Iran to date and confirms that this pathogenic yeast makes a substantial contribution to vaginal infections in this country. However, although significant variations in *C* *africana* prevalence have been reported, between and across several countries worldwide,^12^, ^15^, ^23^ our epidemiological data are in accordance with recent Iranian studies^20^, ^38^ and are quite similar to prevalence rates reported from other single centres in China (6.3%),^50^ Italy (7.2%),^51^ USA (7%)^52^ and Algeria (10%)^53^ indicating that this yeast may be more locally or regionally prevalent.^23^, ^41^ In fact, Turkey, which is geographically close to Iran, showed the lowest prevalence for *C* *africana* with two different studies reporting rates of 0%^54^ and 0.8%,^46^ respectively. Also, other countries, such as Malaysia^55^ and Argentina,^47^, ^48^ reported the lack, or very low prevalence, of *C* *africana* in vaginal samples supporting the hypothesis of local geographical variation, although such estimates may be imprecise due to a limited number of studies in these countries.^23^ However, epidemiological data also revealed that the susceptibility of healthy women to VVC/RVVC, and/or to *Candida* colonisation, could be potentially related to the host genetic background, ethnicity and alteration of the vaginal microbiome.^56^, ^57^ Therefore, it is still unclear whether the differences in *C* *africana* prevalence observed so far^12^, ^15^, ^23^, ^41^ are the result of a limited number of molecular epidemiological studies or whether these differences are somewhat related to geographic/climate variations, race and/or other intrinsic host‐related factors.^8^, ^23^, ^56^, ^57^ For these reasons, further worldwide investigations, using *HWP1* identification method,^31^ should be encouraged in order to assess the real impact of *C* *africana* in VVC/RVVC patients and its global distribution.

Regarding the susceptibility of vaginal *C* *africana* and *C* *dubliniensis* isolates to different antifungal drugs, previous studies showed that these yeasts are generally sensitive to several commonly used antimycotics.^23^, ^35^, ^41^ However, there are some reports showing that *C* *africana* exhibits antifungal susceptibility patterns different from those of *C albicans* ^23^ and some isolates have been classified as resistant to itraconazole, fluconazole, voriconazole, clotrimazole, 5‐flucytosine and terbinafine.^12^, ^22^, ^23^, ^25^

In our study, *C* *africana* and *C* *dubliniensis* isolates showed low MIC values for most of the antifungal drugs tested (Table 2). High MIC values were observed only for NYS and TRB, which is inconsistent with previous reports.^35^, ^47^, ^58^ Furthermore, to our knowledge, we show for the first time a remarkable antifungal activity of EFN (G mean, 0.019 μg/ml) and LUL (G mean, 0.024 μg/ml; Table 2) against all our *Candida* isolates, confirming the excellent in vitro activity of these two new antifungal drugs against a broad spectrum of pathogenic fungi.^59^, ^60^, ^61^, ^62^ However, it should be pointed out that in our study, the susceptibility test was performed using a pH 7 culture medium as described by standard CLSI guidelines.^33^, ^34^ This condition may not reflect the real susceptibility of isolates normally exposed, or adapted, to the vaginal acidic environment (normal pH between 3.8 and 4.5).^63^ Therefore, a sensitivity test with culture medium at pH≃4 should be evaluated in future studies against these vaginal *Candida* pathogens.

In conclusion, although *C* *albicans* remains the main causative agent of vaginal infections in Iranian patients, a considerable part of these cases can be attributed to other *Candida* pathogens that are very difficult to identify using traditional identification methods.^12^, ^39^, ^42^ Nevertheless, based on our data, and several previous studies,^12^, ^23^, ^35^, ^37^, ^41^, ^58^, ^64^ the differentiation between *C* *albicans*, *C* *africana* and *C* *dubliniensis* appears to be clinically irrelevant, as discrimination of these pathogens should not affect therapeutic choices commonly used for the treatment of VVC/RVVC. However, several drug‐resistant *C* *africana* isolates, or strains with reduced susceptibility to different classes of antimycotics,^12^, ^22^, ^23^ have been recently reported, and we also confirmed the occurrence of isolates with high MIC values for NYS and TRB drugs. Therefore, further epidemiological studies on a global scale will be useful to establish the real prevalence, and the role, of *C* *africana* in vaginal infections as well as understanding the extent of antifungal resistance in this pathogenic yeast.

## CONFLICT OF INTEREST

The authors declare that there are no conflicts of interest.

## AUTHOR CONTRIBUTIONS

**Gholamreza Shokoohi:** Conceptualization (lead); Funding acquisition (lead); Supervision (lead); Writing – original draft (lead). **Javad Javidnia:** Formal analysis (lead); Writing – review and editing (equal). **Hossein Mirhendi:** Conceptualization (supporting); Writing – review and editing (equal). **Athar Rasekh Jahromi:** Resources (equal); Writing – review and editing (equal). **Ali Rezaei – Matehkolaei:** Writing – review and editing (equal). **Saham Ansari:** Formal analysis (equal); Writing – review and editing (equal). **Faeze Maryami:** Resources (equal); Writing – review and editing (equal). **Sahand Goodarzi:** Resources (equal); Writing – review and editing (equal). **Orazio Romeo:** Supervision (supporting); Writing – original draft (supporting); Writing – review and editing (lead).

## Data Availability

The data that support the findings of this study are openly available in the Genbank database at https://www.ncbi.nlm.nih.gov/genbank, reference number MT361747–MT361763.
